# Cardiovascular risk factors among ART‐experienced people with HIV in South Africa

**DOI:** 10.1002/jia2.25274

**Published:** 2019-04-16

**Authors:** Emily P Hyle, Linda‐Gail Bekker, Emily B Martey, Mingshu Huang, Ai Xu, Robert A Parker, Rochelle P Walensky, Keren Middelkoop

**Affiliations:** ^1^ Medical Practice Evaluation Center Massachusetts General Hospital Boston MA USA; ^2^ Division of Infectious Diseases Massachusetts General Hospital Boston MA USA; ^3^ Harvard University Center for AIDS Research (CFAR) Boston MA USA; ^4^ Harvard Medical School Boston MA USA; ^5^ Desmond Tutu HIV Centre Institute of Infectious Disease & Molecular Medicine and Department of Medicine Faculty of Health Sciences University of Cape Town Cape Town South Africa; ^6^ Biostatistics Center Massachusetts General Hospital Boston MA USA; ^7^ Division of Infectious Diseases Brigham and Women's Hospital Boston MA USA; ^8^ Division of General Internal Medicine Massachusetts General Hospital Boston MA USA

**Keywords:** HIV, South Africa, cardiovascular, hypertension, diabetes

## Abstract

**Introduction:**

People with HIV (PWH) are at increased risk for atherosclerotic cardiovascular disease (CVD). Screening for CVD risk factors is recommended but not routine in South African HIV clinics. We sought to describe the prevalence of CVD risk factors among antiretroviral treatment (ART)‐experienced patients in South Africa.

**Methods:**

We performed a prospective, observational cross‐sectional study of PWH (>21 years, excluding pregnant women) on ART in South Africa. We interviewed patients regarding CVD risk factors, and obtained two blood pressure (BP) measurements and random/fasting glucose via a point‐of‐care glucometer. Standardized chart reviews provided individuals' HIV‐specific data. We defined hypertension as: self‐reported use of antihypertensives or mean systolic BP (SBP) ≥140 mmHg or diastolic BP (DBP) ≥90 mmHg (Stage 1) or SBP ≥160 mmHg or DBP ≥100 mmHg (Stage 2). We defined diabetes as self‐reported use of insulin/oral hypoglycaemics or fasting (random) glucose ≥7.0 (≥11.1) mmol. We obtained risk ratios (RR) for hypertension from a multivariable log‐binomial regression model, adjusting for age, sex and diabetes.

**Results:**

From March 2015 to February 2016, 458 participants enrolled with median age 38 years (interquartile range (IQR) 33 to 44 years) and median CD4 466/μL (IQR 317 to 638/μL); 78% were women. Participants were on ART for a median of four years, with 33% on ART ≥6 years. Almost a quarter (106/458) met the study definition for hypertension, of whom 45/106 (42%) were previously diagnosed, 23/45 (51%) were on medication and 4/23 (17%) were controlled. Eight participants had asymptomatic hypertensive urgency (BP≥180/110 mmHg). Of the 458 participants, 26 (6%) met the study definition for diabetes, half of whom (13/26) were already diagnosed; 11/13 (85%) were on treatment, of whom 4/11 (36%) had normal glucose. Age was the only significant predictor of hypertension (RR, 1.04; 95% CI, 1.03 to 1.06, *p *< 0.0001) in the multivariable model.

**Conclusions:**

Hypertension and diabetes were prevalent among PWH prescribed ART in South Africa with less than half diagnosed, and still fewer treated and controlled. Hypertension was independently associated with age but not with HIV‐specific factors. Screening for and treatment of CVD risk factors could decrease future morbidity and mortality, especially as this population ages.

## 1 Introduction

More than half of the 7.2 million people with HIV (PWH) in South Africa are on antiretroviral treatment (ART), a testament to dedicated efforts to expand HIV diagnosis and treatment 1. ART has substantially increased life expectancy, and PWH in South Africa who initiate ART before CD4 counts fall below 200/μL can expect a near‐normal life expectancy 2, 3, 4, 5. Virologically suppressed on ART, PWH are now experiencing non‐communicable diseases (NCDs) previously largely associated with ageing 6. Now is the time to consider available opportunities to reduce risk for NCDs and improve overall health.

In recent years, NCDs have emerged as the leading cause of morbidity and mortality among PWH on ART 7, 8, 9. Although certain behavioural risk factors, such as obesity, smoking and nutrition, may contribute to a higher prevalence of NCDs in PWH, people on ART also experience immune activation that is associated with higher risk of NCDs 10, 11. As more PWH are on ART and live near‐normal life spans, NCDs are likely to cause proportionally greater morbidity and mortality 7, 12.

Among the diversity of NCDs facing the ageing population of PWH, cardiovascular disease (CVD) offers specific opportunities for risk reduction because of well‐characterized CVD risk factors that are amenable to screening and treatment 13. In the general population of South Africa, the prevalence of CVD risk factors continues to rise rapidly, creating a major public health concern for the region 14, 15, 16. Hypertension, in particular, is a major cause of morbidity and mortality in South Africa, where more than 60% of those over 50 live with hypertension 17.

The synergy of an increasing prevalence of CVD risk factors among the general population, the ageing of PWH and the increased chronic inflammation experienced by PWH suggests that CVD is likely to become more common among PWH in South Africa. Such a trend would lead to increased morbidity, mortality and costs of care. As the prevalence of CVD risk factors among PWH rises in South Africa and other low‐ and middle‐income countries, integration and implementation of chronic disease management within the HIV care continuum becomes critical to sustain the benefits achieved with ART.

Integration of HIV clinical care with chronic disease management, such as hypertension and diabetes, is a public health priority in South Africa 18. Current guidelines recommend that PWH in South Africa receive blood pressure monitoring and annual screening for diabetes 19, 20. However, screening and clinical management of hypertension, diabetes and other CVD risk factors among PWH in South Africa occur less frequently than recommended, despite a high prevalence of disease 21. Most published data characterize CVD risk factors among severely immunosuppressed PWH or PWH with limited if any ART experience 22, 23, 24, 25. We sought to characterize the prevalence of CVD risk factors, the cascade of hypertension and diabetes care, and any associations with hypertension among ART‐experienced PWH in South Africa.

## 2 Methods

### 2.1 Study design, setting and participants

We performed a prospective, observational cross‐sectional study among a convenience sample of PWH receiving ART at a local community health centre, which has served an urban township of approximately 20,000 people near Cape Town, South Africa since 2004. The clinic provides HIV, family planning and tuberculosis services to the community; 1767 PWH were in care and prescribed ART in 2015. Patients were eligible to participate if they were living with HIV, currently prescribed ART, older than 21 years and not pregnant.

A dedicated study research assistant (RA) offered participation in the study to a convenience sample of patients after they had collected their ART at the pharmacy in the clinic or if they came to clinic requesting participation. The RA obtained formal, written informed consent in English or Xhosa, depending on the participant's preference.

### 2.2 Data collection

#### 2.2.1 CVD‐specific clinical factors

After obtaining informed consent, the RA interviewed the participant in English or Xhosa using a standardized form adapted from the World Health Organization (WHO)'s Stepwise approach to Surveillance (STEPS) instrument and including questions regarding the participant's demographics, self‐reported CVD risk factors, including tobacco use, and any prior diagnosis or treatment of hypertension or diabetes 26.

Data were collected regarding weight, blood pressure and glucose. The RA weighed each patient on a scale. Blood pressures were measured when the participant was in a seated position with legs uncrossed and on the ground. Two blood pressure measurements were obtained at least five minutes apart, and a large cuff was used when needed. Point‐of‐care blood glucose was tested using Glucoplus^®^. Depending on the participant's response to the question, “how many hours ago was your last meal?,” glucose values were considered fasting (≥8 h) or random (<8 h).

#### 2.2.2 HIV‐specific clinical factors

A research nurse used a standardized form to extract data from each participant's clinical chart regarding the patient's height and HIV‐specific data, including the date of HIV diagnosis and treatment history.

### 2.3 Definitions of hypertension and diabetes

We defined hypertension by two parameters: 1) self‐reported use of antihypertensive medications or 2) mean blood pressure ≥140/90 mmHg as measured at the interview, according to WHO guidelines 27. We further classified hypertension as either Stage 1 (systolic blood pressure (SBP) of 140 to 159 mmHg or diastolic blood pressure (DBP) of 90 to 99 mmHg), Stage 2 (SBP ≥160 mmHg or DBP ≥100 mmHg) 27 or hypertensive urgency (SBP ≥180 mmHg or DBP ≥110 mmHg) 19.

We defined diabetes by three parameters: (1) self‐reported use of insulin or oral hypoglycaemic medications or (2) a fasting blood glucose ≥7.0 mmol/L or (3) a random blood glucose level ≥11.1 mmol/L, according to WHO guidelines 28. We considered hyperglycaemia to be severe if blood glucose was >22 mmol/L 29.

To provide conservative disease estimates, participants who self‐reported a prior diagnosis of hypertension or diabetes did not meet our definition unless they stated that they were currently taking medications or had a blood pressure or glucose measurement that met WHO criteria. In a sensitivity analysis, we examined the impact of the more conservative criteria for hypertension endorsed by the American College of Cardiology and American Heart Association (ACC/AHA) in 2017: Stage 1 (SBP of 130 to 149 mmHg or DBP of 80 to 89 mmHg), Stage 2 (SBP ≥140 mmHg or DBP ≥90 mmHg) 30.

### 2.4 Data management and statistical analysis

Study data were double‐entered by the RA or research nurse using REDCap (Research Electronic Data Capture) tools hosted at Massachusetts General Hospital 31. We summarized data as counts and percentages for categorical variables and as medians and interquartile ranges (IQR) for continuous variables. We calculated percentages based on the number of responses per question, excluding any missing responses. We used multivariable log**‐**binomial regression to identify associations with hypertension and considered the following covariates: age, sex, current CD4 cell count, nadir CD4 cell count (i.e. lowest CD4 recorded), total time on ART, type of ART, diabetes and tobacco use. In the final multivariable model, we included only covariates that were significantly associated (*p* < 0.05) with hypertension in the univariate models; we also included diabetes in the multivariable model because of biologic plausibility. There were an insufficient number of diabetic participants in the sample to perform multivariable regression to identify associations with diabetes. We described associations with relative risk, 95% confidence intervals and *p*‐values. Two‐tailed *p*‐values of less than 0.05 were considered to indicate statistical significance. Statistical analyses were performed with the use of SAS software, version 9.4 (SAS Institute).

### 2.5 Ethics approvals

The study protocol was reviewed and approved by Human Research Ethics Committee at University of Cape Town (575/2014) and by the Institutional Review Board at Massachusetts General Hospital (2014P001661/PHS).

## 3 Results

### 3.1 Cohort demographics

**Figure 1:**
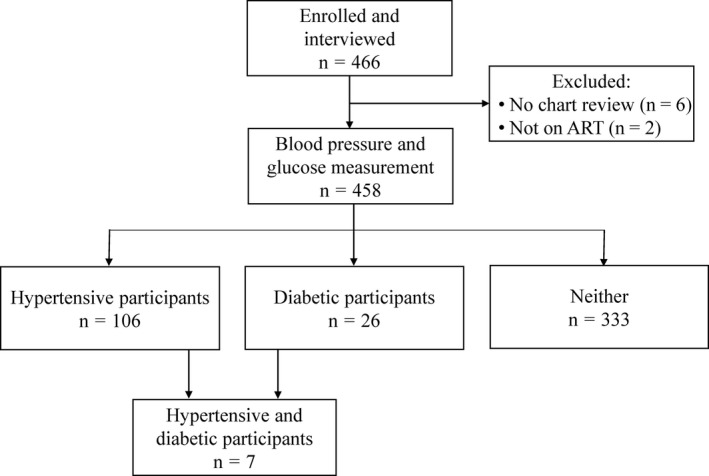
Flow diagram of study participants enrolment and participation Of the 466 participants enrolled, 6 participants were excluded because their clinical charts were not available for review by a research nurse, and two participants were excluded because they were not taking ART. Of the 458 participants, 106 participants were hypertensive and 26 participants were diabetic. Seven participants were both hypertensive and diabetic.

Of the 1767 patients receiving ART care at this site in 2015, 466 participants enrolled between March 2015 and February 2016 after giving informed consent (Figure 1). Six participants were later excluded because they did not have data available from chart review. Two participants were excluded because they were not taking ART.

Of 458 participants included in the study, 356 (78%) were female with a median age of 38 years (IQR 33 to 44 years) (Table 1, left). The median current CD4 cell count was 466 cells/μL (IQR 317 to 638 cells/μL), and the median reported nadir CD4 cell count was 205 cells/μL (IQR 122 to 312 cells/μL). Participants had spent a median time on ART of four years; 153 participants (33%) had been prescribed ART for more than six years. Nearly 90% of the participants were prescribed a non‐nucleoside reverse transcriptase inhibitor‐based or combination regimen; 60 participants (13%) were prescribed a PI‐based regimen. Three hundred and eighty‐one participants (83%) were continuously in care and prescribed ART as per clinic records. Of the 208 participants with available pill counts (the only available adherence estimate), 193 (93%) demonstrated greater than 90% adherence to ART. Nearly half of the participants (42%) were previously treated for TB.

**Table 1:** Cohort demographics, HIV disease and treatment specifics, and CVD risk factors among PWH prescribed ART

Covariate	All participants (n=458)^a^	Participants with hypertension (n=106)^b^	Participants with diabetes (n=26)^c^
Sex (female, n (%))	356 (78%)	71 (67%)	22 (85%)
Age (years, median (IQR))	38 (33 to 44)	44 (38 to 47)	43 (38 to 52)
Race (Black African, n (%))	448 (98%)	101 (95%)	23 (88%)
Monthly **i**ncome (rand, median, (IQR))	2400 (1440 to 3500)	2000 (1450 to 3500)	2000 (1450 to 3000)
HIV disease and treatment specifics			
Current CD4 cell count (/μL, median (IQR))	466 (317 to 638)	458 (315 to 631)	673 (316 to 774)
Nadir CD4 cell count (/μL, median (IQR))	205 (122 to 312)	210 (147 to 312)	173 (107 to 351)
Time on ART (years, median (IQR))	4 (2 to 7)	5 (2 to 8)	5 (3 to 8)
Type of ART (non‐PI‐based regimen, %)	398 (87%)	96 (91%)	23 (88%)
Type of ART (PI‐based regimen, %)	60 (13%)	10 (9%)	3 (12%)
History of never stopping ART (n, %)	381 (83%)	90 (85%)	21 (81%)
≥90% adherent to ART (n, %)	193 (93%)	47 (94%)	13 (100%)
TB disease and treatment specifics			
Previously treated for TB (n, %)	191 (42%)	43 (41%)	12 (46%)
Currently treated for TB (n, %)	15 (3%)	2 (2%)	1 (4%)
Time on treatment (months, median (IQR))	3 (2 to 5)	2.5 (2 to 3)	5 (5 to 5)
CVD risk factors			
Blood pressure			
Systolic (mmHg, median (IQR))	120 (109 to 132)	140 (134 to 148)	129 (116 to 133)
Diastolic (mmHg, median (IQR))	79 (71 to 86)	93 (90 to 100)	81 (77 to 85)
Blood glucose			
Fasting (mmol/L, median (IQR))	5.2 (4.8 to 5.9)	5.5 (5.2 to 6.0)	8.2 (7.1 to 8.8)
Random (mmol/L, median (IQR))	5.7 (5.2 to 6.4)	5.8 (5.1 to 6.7)	14.2 (10.9 to 20.8)
BMI (kg/m^2^, median (IQR))	27.4 (23.5 to 33.6)	28.8 (24.5 to 34.0)	26.1 (21.6 to 33.8)
Normal/Underweight (n, %)	105 (37%)	25 (36%)	5 (33%)
Overweight (n, %)	76 (27%)	15 (22%)	4 (27%)
Obese (n, %)	102 (36%)	29 (42%)	6 (40%)
Current self‐reported tobacco use (yes, %)	69 (15%)	22 (21%)	6 (23%)
Cigarettes smoked daily			
<3 cigarettes (n, %)	15 (22%)	1 (5%)	1 (17%)
3 to 5 cigarettes (n, %)	32 (46%)	15 (68%)	4 (67%)
6 to 8 cigarettes (n, %)	3 (4%)	2 (9%)	0 (0%)
10+ cigarettes (n, %)	18 (26%)	4 (18%)	1 (17%)

ART, antiretroviral therapy; BMI, body mass index; CVD, cardiovascular disease; IQR, interquartile range; PI, protease inhibitor.

^a^Data are missing for the following covariates: income (69), current and nadir CD4 (5), time on ART (31), never stopped taking ART (3), adherence to ART (250), previously treated for TB (2) fasting and random glucose (2), BMI (175) and current self‐reported tobacco use (1); ^b^data are missing for the following covariates: income (16), nadir CD4 (1), time on ART (5), adherence to ART (56), fasting and random glucose (1) and BMI (37); ^c^data are missing for the following covariates: income (7), time on ART (2), adherence to ART (13) and BMI (11).

Characteristics of the participants' CVD risk factors included median blood pressure (120 mmHg (IQR 109 to 132 mmHg)/79 mmHg (IQR 71 to 86 mmHg)), median fasting glucose (5.2 mmol/L (IQR 4.8 to 5.9 mmol/L)) and median random glucose (5.7 mmol/L (IQR 5.2 to 6.4 mmol/L)). The median body mass index (BMI) was 27.4 kg/m^2^ (IQR 23.5 to 33.6 kg/m^2^), with 102 participants (36%) meeting criteria for obesity (BMI>30 kg/m^2^). Sixty‐nine participants (15%) reported current tobacco use, of whom 32 (46%) smoked 3 to 5 cigarettes/daily and 18 (26%) smoked 10 or more cigarettes/daily.

### 3.2 Hypertensive participants

Of the 458 participants, 106 (23%) met the study definition of hypertension, including 102 individuals with elevated blood pressure and four individuals with normal blood pressure who reported being on antihypertensive medications.

**Figure 2:**
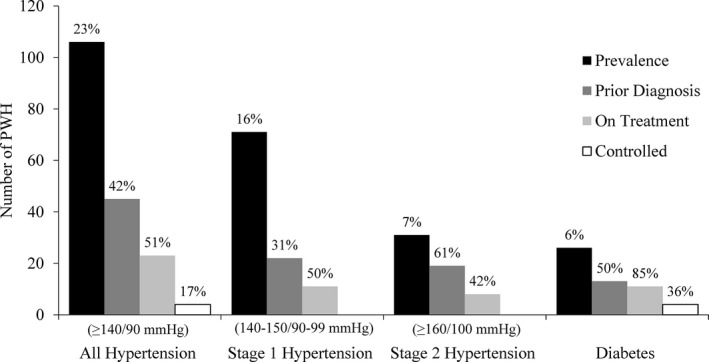
Cascade of care for hypertension and diabetes among people with HIV on ART A total of 106 participants met the study definition of hypertension, of whom 45 (42%) reported a prior diagnosis and 23 (51%) were currently on antihypertensive medication. Only 4 (17%) participants taking antihypertensive medication had a controlled blood pressure. Among the participants with high blood pressure at the interview, 16% had Stage 1 hypertension and 7% had Stage 2 hypertension. The overall prevalence of diabetes was 6%, of whom 50% were aware of their diabetes and 85% of whom were on diabetes treatment. Of the 11 participants on diabetes treatment, 4 (36%) had controlled blood glucose levels during the interview. The prevalence percentage is calculated over all participants; the percentages of prior diagnosis, on treatment and controlled patients are based on the prevalence of prior group in the cascade.

Of the 106 hypertensive participants, 45 (42%) had a self‐reported, prior diagnosis of hypertension; 23 (51%) of the previously diagnosed were currently on treatment with antihypertensive medications (Figure 2, left). Only four (17%) of those prescribed antihypertensives had blood pressure in normal range; the severity of underlying hypertension could not be assigned to these patients as their blood pressures were normal when tested while taking medications. Seventy‐one participants (16%) had Stage 1 hypertension, and 31 (7%) had Stage 2 hypertension. A greater proportion of those with Stage 2 hypertension knew of their diagnosis (Stage 1 hypertension, 31% previously diagnosed; Stage 2 hypertension, 61% previously diagnosed). However, participants with Stage 2 hypertension were not more likely to be on treatment (Stage 1 hypertension, 50%; Stage 2 hypertension, 42%). Using the more stringent 2017 ACC/AHA definition of hypertension, more than half (51%) of the study population had blood pressures that met criteria for hypertension, including 131 participants (29%) with Stage 1 hypertension and 102 (22%) with Stage 2 hypertension (Figures S1 and S2).

Cohort characteristics of all hypertensive participants are outlined in Table 1 (middle column). Hypertensive participants were notably older compared with the entire population (median age 44 years compared to 38 years), more often male (33% compared to 22%) and more likely to be smokers (21% compared to 15%); other variables were similar in the two groups. The median blood pressure of the hypertensive participants was 140 mmHg (IQR 134 to 148 mmHg)/93 mmHg (IQR 90 to 100 mmHg)). Comparisons of participants with hypertension given the 2017 ACC/AHA guidelines are included in Table S1.

Eight (8%) participants had hypertension urgency (median blood pressure, 162 mmHg (IQR 140 to 187 mmHg)/111 mmHg (IQR 110 to 122 mmHg)). Of these, two (25%) were new diagnoses, and six (75%) had a prior history of hypertension of whom four (67%) were already prescribed antihypertensive medications. None were symptomatic.

### 3.3 Diabetic participants

Twenty‐six (6%) participants met study criteria for diabetes. Of these 26 participants, 13 (50%) reported a prior diagnosis, of whom 11 (85%) were currently on treatment (Figure 2, right). Of the eleven participants already prescribed treatment for their diabetes, four (36%) had blood glucose in the normal range when measured during the interview.

The diabetic participants had similar characteristics to the general study population except for age (43 years compared to 38 years) and current CD4 cell count (673/μL compared to 466/μL) (Table 1, right column). The median fasting glucose of these participants was 8.2 mmol/L (IQR 7.1 to 8.8 mmol/L), and the median random glucose was 14.2 mmol/L (IQR 10.9 to 20.8 mmol/L). Of the 26 participants with diabetes, only one previously diagnosed participant (4%) had severe hyperglycaemia (random glucose >22 mmol/L) but was not symptomatic. The participant was not taking medications for diabetes, despite the known diagnosis.

### 3.4 Multiple CVD risk factors

More than half of these ART‐experienced PWH (51%) had one or more CVD risk factors evident on this one‐time screening interview. One hundred and seventy‐three (38%) participants had one CVD risk factor, whereas fifty‐four (12%) participants had two CVD risk factors and only seven (2%) participants had three or more CVD risk factors. When the more stringent 2017 ACC/AHA guidelines for hypertension were applied, only 150 (33%) participants had no CVD risk factors; 198 (43%) participants had one CVD risk factor, 98 (21%) participants had two and 12 (3%) participants had three or more. These estimates are likely conservative given that BMI data were missing on 175 participants.

### 3.5 Associations of hypertension

We performed multivariable log‐binomial regression to assess possible associations with hypertension. BMI was not included in the model given the extent of missing data. Current or nadir CD4 cell count, time on ART, PI‐based ART, diabetes and tobacco use were not independently associated with hypertension; of these, only diabetes was included in the final model because of its biologic plausibility (Table 2 and Table S2 with the 2017 ACC/AHA guidelines). Our analysis found that age was associated with a diagnosis of hypertension in this cohort: there was a 4% increase in the relative risk of having a hypertension diagnosis for every additional year of age.

**Table 2:** Log‐binomial regression analysis for predictors of hypertension among PWH prescribed ART

Covariate	Unadjusted RR (95% CI)	*p*‐value for unadjusted RR	Adjusted RR (95% CI)	*p*‐value for adjusted RR
Age (years)	1.05 (1.03 to 1.06)	<0.0001	1.04 (1.03 to 1.06)	<0.0001
Sex (female)	0.58 (0.41 to 0.82)	0.002	0.83 (0.58 to 1.18)	0.29
**HIV disease and treatment specifics**				
Current CD4 (cells/μL)				
>500	REF	0.92		
350 to 500	1.21 (0.80 to 1.83)			
200 to 349	1.17 (0.75 to 1.82)			
100 to 199	1.17 (0.56 to 2.46)			
50 to 99	1.17 (0.48 to 2.85)			
<50	0.67 (0.11 to 4.18)			
Nadir CD4 (cells/μL)				
>500	REF	0.39		
350 to 500	2.02 (0.62 to 6.62)			
200 to 349	2.45 (0.82 to 7.36)			
100 to 199	2.17 (0.72 to 6.56)			
50 to 99	1.76 (0.53 to 5.88)			
<50	1.54 (0.42 to 5.61)			
Total time on ART (years)				
<2	REF	0.62		
2 to 4	1.16 (0.70 to 1.91)			
4 to 6	0.96 (0.51 to 1.81)			
>6	1.29 (0.82 to 2.03)			
On PI‐based ART (yes)	0.69 (0.38 to 1.25)	0.22		
**CVD risk factors**				
Diabetes (yes)	1.17 (0.61 to 2.27)	0.63	0.91 (0.50 to 1.65)	0.75
Self‐reported tobacco use (yes)	1.48 (1.00 to 2.19)	0.052		

ART, antiretroviral therapy; CI, confidence interval; CVD, cardiovascular disease; PI, protease inhibitor; RR, relative risk.

## 4 Discussion

CVD risk factors were prevalent among a population of PWH highly adherent to ART in South Africa for a median of four years. Hypertension and diabetes were underdiagnosed, undertreated and poorly controlled despite frequent follow‐up in HIV clinical care. Although hypertension was evident in almost a quarter of these ART‐experienced participants, hypertension was not independently associated with ART duration, PI use, current or nadir CD4 cell count.

Our findings are consistent with previous reports of CVD risk factor prevalence among ART‐experienced PWH in South Africa: hypertension (12% to 39%) 6, 32, 33, 34, 35, 36, 37; Stage 2 hypertension (14% to 66%) 32, 38; impaired glucose tolerance (16% to 21%) 22, 23, 39, 40, 41, 42; diabetes (2% to 8%) 23, 36, 40, 42, 43; and self‐reported tobacco smoking (1% to 31%) 44, 45, 46, 47, 48. This substantial prevalence of CVD risk factors among a patient population who attends clinic on a routine basis underscores an important opportunity to screen for these intervenable risk factors. Identification and management of CVD risk factors could reduce future risk of highly morbid and costly CVD‐associated diseases such as stroke, myocardial infarction and peripheral arterial disease 13, 49. Additionally, our study population had a high prevalence and number of CVD risk factors, despite a mean age of only 38 years. As patients continue to grow older on ART, a further increase in the prevalence and severity of CVD risk factors can be expected, increasing the need for screening and management of these modifiable risk factors.

This study is the first to our knowledge that reports on the substantial number of patients with asymptomatic hypertensive urgency. Eight asymptomatic participants (~2%) attending a routine clinical visit for HIV clinical care had a mean blood pressure measurement that met criteria for urgent management by the South African Guidelines for Hypertension Management 19. Of these participants, 75% were already aware of their hypertension diagnosis yet still had severely uncontrolled blood pressure, which underscores the need for improved medical management of this chronic disease that can also provoke acute illness. An additional 25% of these participants denied a prior diagnosis and thus likely only learned of their hypertension diagnosis from screening given participation in this study, emphasizing the immediate clinical impact that screening could offer in these settings.

This study results underscore the leaky clinical care cascade of non‐communicable diseases that is experienced in many low‐ and middle‐income countries 50. Among participating participants in this study, 42% were aware of a past diagnosis of hypertension, of whom 51% were on treatment; however, only 17% of patients prescribed antihypertensives had blood pressures in normal range. Data from Uganda and Tanzania demonstrate the same leaky care cascade: only 20% to 25% of patients with hypertension who attended HIV clinics were already aware of their diagnosis, and only 14% were on treatment with roughly half under control 38, 51. In our study population, 50% of participants who met criteria for diabetes were already diagnosed and 85% were on treatment with 36% in control; these findings are similar to those reported from South Africa regarding the diabetes care cascade among the participants not living with HIV (40% diagnosed; 94% on treatment; 51% in control) 52. Efforts to improve the HIV care cascade should be leveraged to improve the care cascade for hypertension and diabetes, but these opportunities also must be balanced with the risks of overburdening clinics, especially given the potential for increased clinical services leading to longer wait times and patient attrition 53, 54.

These results support previously published studies that hypertension is associated with age but not with HIV‐specific risk factors. Most studies have described an association between hypertension and older age, male sex and elevated body mass index but not with HIV‐specific risk factors 6, 33, 35, 38, 55, 56. Two studies have described an association between incident hypertension and ART exposure 57 or nadir CD4 58, and data are emerging regarding the association between tuberculosis and CVD risk 59. Because early initiation of ART is now recommended for all PWH given its clinical and prevention benefits, the exact relationship between immunosuppression, ART exposure and hypertension incidence may be less relevant as all patients will be encouraged to start ART upon diagnosis 60.

Our study has limitations. Some participants were missing data, especially on weight, which limited our ability to calculate BMI and to examine the impact of obesity. Additionally, data were not available on dyslipidaemia or viral load. Screening was performed using point‐of‐care glucose instead of oral glucose tolerance testing 20, and participants could have been misclassified as “fasting” if they did not disclose recent snacks or sugary drinks when asked about recent meals. We were conservative in our definitions of hypertension and diabetes by excluding the 57 participants who offered a self‐reported prior diagnosis of hypertension (and the six with self‐reported diabetes) but denied taking medications and had normal values on screening. Multiple measurements on different days could not be obtained to confirm a diagnosis of hypertension or diabetes because of uncertainty regarding when participants would return to clinic; therefore, participants could be miscategorized without confirmatory testing, especially if their blood pressure or glucose measurements were near the diagnostic threshold. Data were collected at a single clinical site as a convenience sample so that participants may not be fully representative of the clinic population. Although the demographics of our convenience sample were not substantially dissimilar from the overall clinic population, the study population included a greater proportion of females (clinic: 71% female; study: 78% female) and older patients (median age in clinic: 32 years; study: 38 years). Given that hypertension and diabetes are associated with older age, our data may overestimate these CVD risk factors among all PWH in South Africa but may reflect the ART‐experienced population. Results might not be generalizable to other settings within South Africa or sub‐Saharan Africa.

## 5 Conclusions

In conclusion, hypertension, diabetes, smoking and obesity were common among highly adherent, ART‐experienced PWH at an HIV clinic in South Africa. These patients are likely at increased lifetime risk of CVD and further attention should be paid to screening for intervenable CVD risk factors and opportunities to integrate care services for PWH.

## Competing interests

The authors report no competing interests.

## Authors' contributions

EPH, RAP and KM designed the study. EPH wrote the first draft of the manuscript. EPH, LGB, EBM, MH, AX, RAP, RPW and KM provided critical revisions of the manuscript. All authors have read and approved the final version of the paper.

## Supporting information


**Table S1.** Cohort demographics, HIV disease and treatment specifics, and CVD risk factors among PWH prescribed ART using 2017 ACC/AHA definition of hypertension
**Table S2.** Log‐binomial regression analysis for predictors of hypertension among PWH prescribed ART using 2017 ACC/AHA definition of hypertension

**Figure S1.** Flow diagram of study participants enrolment and participation using the American Academy of Cardiology/American Heart Association hypertension guidelines.

**Figure S2.** Cascade of care for hypertension among PWH on ART using the 2003 World Health Organization/International Society of Hypertension and the 2017 American Academy of Cardiology/American Heart Association hypertension guidelines.
