# Supplementary figures and images for: Cardiovascular risk factors among ART‐experienced people with HIV in South Africa
Source: J Int AIDS Soc. 2019 Apr 16;22(4):e25274. doi: 10.1002/jia2.25274 (PMC6466898; doi:10.1002/jia2.25274)

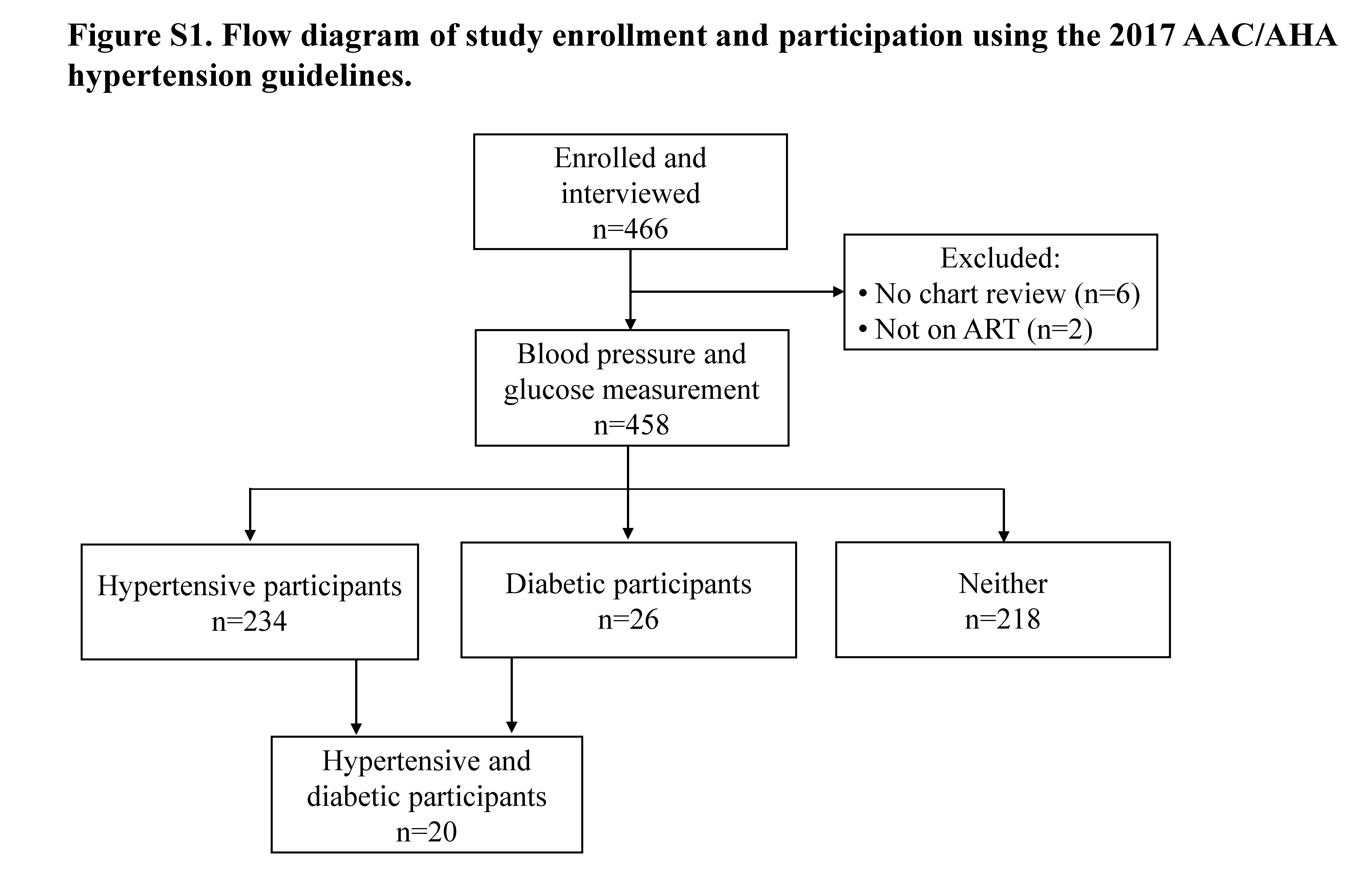

Supplement: Supplementary file 1 — Table S1. Cohort demographics, HIV disease and treatment specifics, and CVD risk factors among PWH prescribed ART using 2017 ACC/AHA definition of hypertension Table S2. Log‐binomial regression analysis for predictors of hypertension among PWH prescribed ART using 2017 ACC/AHA definition of hypertension [file JIA2-22-e25274-s001.png]

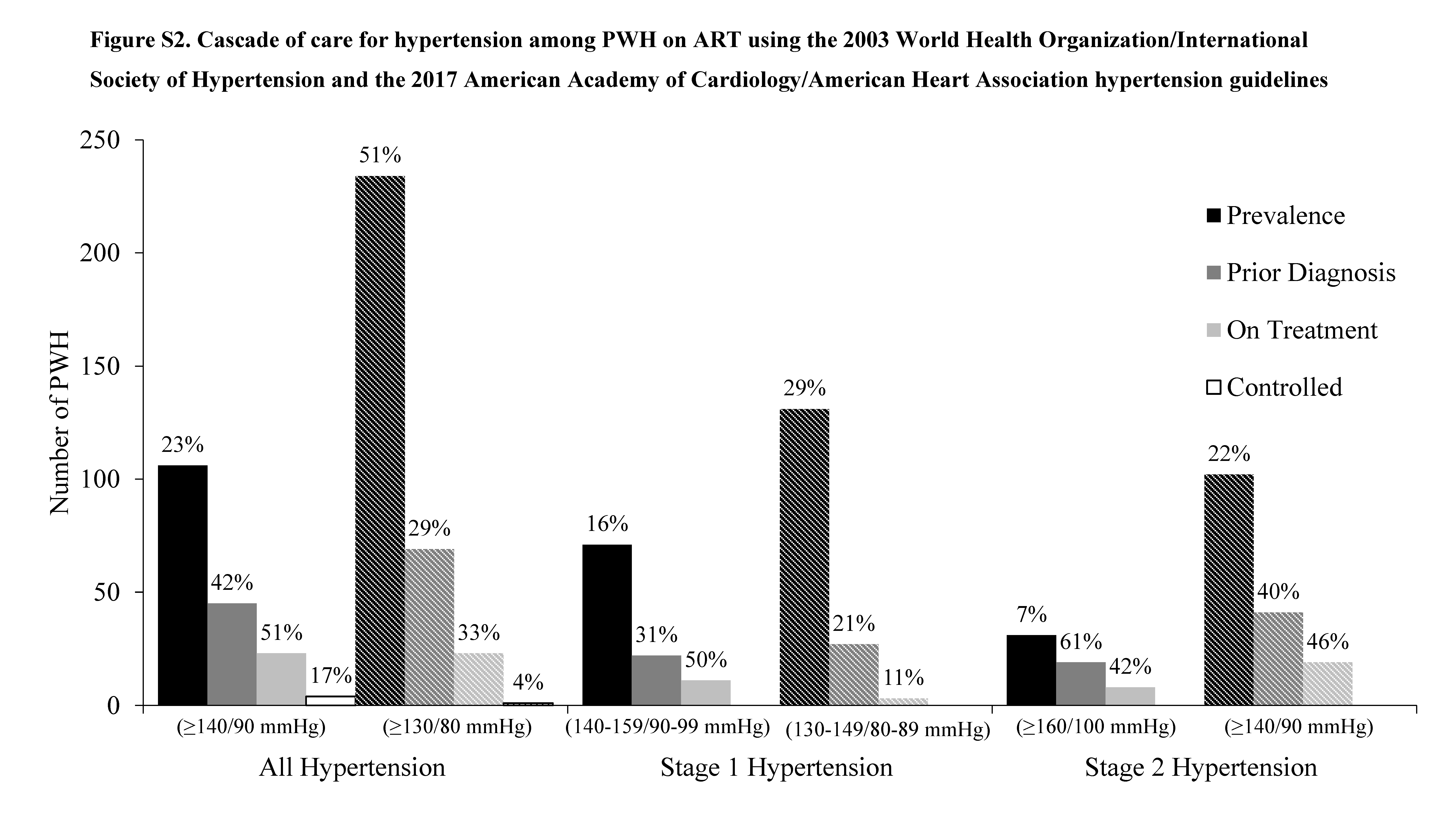

Supplement: Supplementary file 3 — Figure S2. Cascade of care for hypertension among PWH on ART using the 2003 World Health Organization/International Society of Hypertension and the 2017 American Academy of Cardiology/American Heart Association hypertension guidelines. [file JIA2-22-e25274-s003.png]
