# Supplementary material for: Cardiovascular risk factors among ART‐experienced people with HIV in South Africa
Source: J Int AIDS Soc. 2019 Apr 16;22(4):e25274. doi: 10.1002/jia2.25274 (PMC6466898; doi:10.1002/jia2.25274)
Supplement: Supplementary file 2 — Figure S1. Flow diagram of study participants enrolment and participation using the American Academy of Cardiology/American Heart Association hypertension guidelines. [file JIA2-22-e25274-s002.docx]

**Table S1.** Cohort demographics, HIV disease and treatment specifics, and CVD risk factors among PWH prescribed ART using 2017 ACC/AHA definition of hypertension

| **Covariate** | **All participants**  **(n=458)*** | **Participants with hypertension (n=234)**** | **Participants with diabetes**  **(n=26)***** |
| --- | --- | --- | --- |
| Sex (female, n (%)) | 356 (78%) | 169 (72%) | 22 (85%) |
| Age (years, median (IQR)) | 38 (33-44) | 42 (35-47) | 43 (38-52) |
| Race (black African, n (%)) | 448 (98%) | 225 (96%) | 23 (88%) |
| Monthly income (rand, median, (IQR)) | 2400 (1440-3500) | 2400 (1450-3500) | 2000 (1450-3000) |
| **HIV Disease and Treatment Specifics** |  |  |  |
| Current CD4 cell count (/μL, median (IQR)) | 466 (317-638) | 455 (309-628) | 673 (316-774) |
| Nadir CD4 cell count (/μL, median (IQR)) | 205 (122-312) | 199 (124-313) | 173 (107-351) |
| Time on ART (years, median (IQR)) | 4 (2-7) | 4 (2-8) | 5 (3-8) |
| Type of ART (non-PI-based regimen, %) | 398 (87%) | 211 (90%) | 23 (88%) |
| Type of ART (PI-based regimen, %) | 60 (13%) | 23 (10%) | 3 (12%) |
| History of never stopping ART (n, %) | 381 (83%) | 193 (82%) | 21 (81%) |
| ≥90% adherent to ART (n, %) | 193 (93%) | 106 (95%) | 13 (100%) |

**Table S1.** Cohort demographics, HIV disease and treatment specifics, and CVD risk factors among PWH prescribed ART using 2017 ACC/AHA definition of hypertension (continued)

| **Covariate** | **All participants**  **(n=458)*** | **Participants with hypertension (n=234)**** | **Participants with diabetes**  **(n=26)***** |
| --- | --- | --- | --- |
| **TB Disease and Treatment Specifics** |  |  |  |
| Previously treated for TB (n, %) | 191 (42%) | 99 (42%) | 12 (46%) |
| Currently treated for TB (n, %) | 15 (3%) | 8 (3%) | 1 (4%) |
| Time on treatment (months, median (IQR)) | 3 (2-5) | 2.5 (2-4) | 5 (5-5) |
| **CVD Risk Factors** |  |  |  |
| Blood pressure |  |  |  |
| Systolic (mmHg, median (IQR)) | 120 (109-132) | 131 (123-140) | 129 (116-133) |
| Diastolic (mmHg, median (IQR)) | 79 (71-86) | 86 (81-93) | 81 (77-85) |
| Blood glucose |  |  |  |
| Fasting (mmol/L, median (IQR)) | 5.2 (4.8-5.9) | 5.4 (5.0-6.0) | 8.2 (7.1-8.8) |
| Random (mmol/L, median (IQR)) | 5.7 (5.2-6.4) | 5.8 (5.2-6.5) | 14.2 (10.9-20.8) |

**Table S1.** Cohort demographics, HIV disease and treatment specifics, and CVD risk factors among PWH prescribed ART using 2017 ACC/AHA definition of hypertension (continued)

| **Covariate** | **All participants**  **(n=458)*** | **Participants with hypertension**  **(n=234)**** | **Participants with diabetes**  **(n=26)***** |
| --- | --- | --- | --- |
| BMI (kg/m^2^, median (IQR)) | 27.4 (23.5-33.6) | 28.1 (24.1-35.1) | 26.1 (21.6-33.8) |
| Normal/Underweight (n, %) | 105 (37%) | 50 (36%) | 5 (33%) |
| Overweight (n, %) | 76 (27%) | 33 (24%) | 4 (27%) |
| Obese (n, %) | 102 (36%) | 55 (40%) | 6 (40%) |
| Current self-reported tobacco use (yes, %) | 69 (15%) | 42 (18%) | 6 (23%) |
| Cigarettes smoked daily |  |  |  |
| <3 cigarettes (n, %) | 15 (22%) | 8 (19%) | 1 (17%) |
| 3-5 cigarettes (n, %) | 32 (46%) | 21 (50%) | 4 (67%) |
| 6-9 cigarettes (n, %) | 3 (4%) | 3 (7%) | 0 (0%) |
| 10+ cigarettes (n, %) | 18 (26%) | 10 (24%) | 1 (17%) |

* Data are missing for the following covariates: income (69), current and nadir CD4 (5), time on ART (31), never stopped taking ART (3), adherence to ART (250), previously treated for TB (2) fasting and random glucose (2), BMI (175), and current self-reported tobacco use (1).

** Data are missing for the following covariates: income (43), nadir CD4 (2), time on ART (16), adherence to ART (122), fasting and random glucose (2), and BMI (96).

*** Data are missing for the following covariates: income (7), time on ART (2), adherence to ART (13), and BMI (11).

Abbreviations: IQR: Inter-quartile range; ART, antiretroviral therapy; PI, protease inhibitor; CVD, cardiovascular disease; BMI, body mass index; ACC, American Academy of Cardiology; AHA, American Heart Association

**Table S2.** Log-binomial regression analysis for predictors of hypertension among PWH prescribed ART using 2017 ACC/AHA definition of hypertension

| **Covariate** | **Unadjusted RR (95% CI)** | **p-value for unadjusted RR** | **Adjusted RR (95% CI)** | **p-value for adjusted RR** |
| --- | --- | --- | --- | --- |
| Age (years) | 1.02 (1.02-1.03) | <0.0001 | 1.01 (1.01-1.01) | <0.0001 |
| Sex (female) | 0.74 (0.62-0.89) | 0.0016 | 0.98 (0.87-1.09) | 0.66 |
| **HIV Disease and Treatment Specifics** | | | | |
| Current CD4 (cells/μL) |  | 0.28 |  |  |
| >500 | REF |  |  |  |
| 350-500 | 1.05 (0.83-1.33) |  |  |  |
| 200-349 | 1.26 (1.01-1.57) |  |  |  |
| 100-199 | 1.14 (0.77-1.69) |  |  |  |
| 50-99 | 1.31 (0.88-1.97) |  |  |  |
| <50 | 0.60 (0.18-1.95) |  |  |  |
| Nadir CD4 (cells/μL) |  | 0.88 |  |  |
| >500 | REF |  |  |  |
| 350-500 | 1.30 (0.77-2.20) |  |  |  |
| 200-349 | 1.24 (0.77-2.01) |  |  |  |
| 100-199 | 1.33 (0.82-2.14) |  |  |  |
| 50-99 | 1.25 (0.74-2.12) |  |  |  |
| <50 | 1.19 (0.68-2.10) |  |  |  |

**Table S2.** Log-binomial regression analysis for predictors of hypertension among PWH prescribed ART using ACC/AHA definition of hypertension (continued)

| **Covariate** | **Unadjusted RR (95% CI)** | **p-value for unadjusted RR** | **Adjusted RR (95% CI)** | **p-value for adjusted RR** |
| --- | --- | --- | --- | --- |
| Total time on ART (years) |  | 0.32 |  |  |
| <2 | REF |  |  |  |
| 2-4 | 0.87 (0.66-1.15) |  |  |  |
| 4-6 | 0.90 (0.65-1.25) |  |  |  |
| >6 | 1.08 (0.86-1.36) |  |  |  |
| On PI-based ART (yes) | 0.72 (0.52-1.01) | 0.06 |  |  |
| **CVD Risk Factors** |  |  |  |  |
| Diabetes (yes) | 1.55 (1.23-1.96) | 0.0002 | 1.08 (0.92-1.26) | 0.35 |
| Self-reported tobacco use (yes) | 1.23 (1.00-1.53) | 0.06 |  |  |

Abbreviations: RR, risk ratio; CI, confidence interval; ART, antiretroviral therapy; PI, protease inhibitor; CVD, cardiovascular disease; ACC, American Academy of Cardiology; AHA, American Heart Association.

**FIGURE LEGENDS**

**Figure S1. Flow diagram of study participant enrollment and participation using the American Academy of Cardiology/American Heart Association hypertension guidelines.**

Of the 466 participants enrolled, 6 participants were excluded because their clinical charts were not available for review by a research nurse, and two participants were excluded because they were not taking ART. Of the 458 participants, 234 participants were hypertensive using 2017 ACC/AHA criteria, and 26 subjects were diabetic. Twenty participants were both hypertensive and diabetic.

**Figure S2. Cascade of care for hypertension among PWH on ART using the 2003 World Health Organization/International Society of Hypertension and the 2017 American Academy of Cardiology/American Heart Association hypertension guidelines.**

Using the 2003 World Health Organization/International Society of Hypertension definition of hypertension (solid bars), a total of 106 participants met the study definition of hypertension, of whom 45 (42%) reported a prior diagnosis and 23 (51%) were currently on anti-hypertensive medication. Only 4 (17%) participants taking anti-hypertensive medication had a controlled blood pressure. Among the participants with high blood pressure at the interview, 16% had Stage 1 hypertension and 7% had Stage 2 hypertension. Using the 2017 American College of Cardiology/American Heart Association definition of hypertension (hatched bars), a total of 234 participants met the study definition of hypertension, of whom 69 (29%) reported a prior diagnosis and 23 (33%) were currently on anti-hypertensive medication. One (4%) participant taking anti-hypertensive medication had a controlled blood pressure. Among participants with high blood pressure at the interview, 29% had Stage 1 hypertension and 22% had Stage 2 hypertension. The prevalence percentage is calculated over all participants; the percentages of prior diagnosis, on treatment, and controlled patients are based on the prevalence of prior group in the cascade.
